# Supplementary material for: Clinical Profile, Treatment and Predictors during the First COVID-19 Wave: A Population-Based Registry Analysis from Castile and Leon Hospitals
Source: Int J Environ Res Public Health. 2020 Dec 14;17(24):9360. doi: 10.3390/ijerph17249360 (PMC7765016; doi:10.3390/ijerph17249360)
Supplement: Supplementary file 1 [file ijerph-17-09360-s001.zip › Supplementary material.docx]

**Supplementary Table S1**. Castile and Leon hospital network description

| **Hospital category** | **Hospital denomination** | **Bed capacity** |
| --- | --- | --- |
| Regional | "Santiago Apostol" Hospital | 114 |
|  | "Santos Reyes" Hospital | 123 |
|  | Medina del Campo Hospital | 119 |
| General | Avila Hospital Complex | 430 |
|  | El Bierzo Hospital | 408 |
|  | Palencia Hospital Complex | 495 |
|  | Segovia Hospital Complex | 375 |
|  | Soria Hospital Complex | 319 |
|  | Zamora Hospital Complex | 540 |
| First level | "Rio Hortega" University Hospital | 606 |
|  | Burgos University Hospital Complex | 865 |
|  | Leon University Hospital Complex | 1056 |
|  | Salamanca University Hospital Complex | 914 |
|  | Valladolid University Clinical Hospital | 777 |
